# Supplementary material for: SCFRMF mediates degradation of the meiosis-specific recombinase DMC1
Source: Nat Commun. 2023 Aug 19;14:5044. doi: 10.1038/s41467-023-40799-5 (PMC10439943; doi:10.1038/s41467-023-40799-5)
Supplement: Supplementary file 1 — Supplementary Information [file 41467_2023_40799_MOESM1_ESM.pdf]

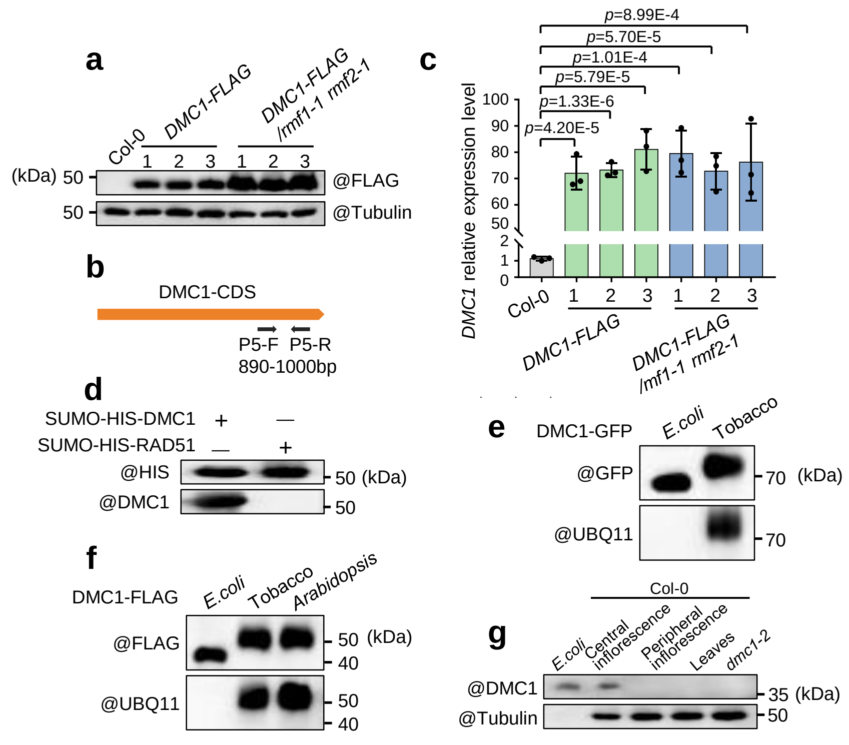

Supplementary Fig. 1. DMC1 can be ubiquitinated *in vivo*.

(a) Western blot shows the expression of DMC1-FLAG in inflorescences of three individual plants from *DMC1-FLAG* and *DMC1-FLAG/rmf1-1 rmf2-1* transgenic lines used in Fig.1a, c, e (second to fourth lanes) and Fig.6a (second to seventh lanes). The *DMC1* CDS sequence (without stop codon) with FLAG was cloned and placed under the control of the *ACT7* promoter. **(b)** Schematic diagram of *DMC1* CDS to show the primer pair used for qRT-PCR in (c) (DMC1-P5) marked below the gene structure as black arrows. **(c)** qRT-PCR shows the relative gene expression level in inflorescences of Col-0 and three biologically independent plants from *DMC1-FLAG* and three biologically independent plants from *DMC1-FLAG/rmf1-1 rmf2-1* transgenic lines used in Fig.1a, c, e (second to fourth panels) and Fig.6a (second to seventh panels) with the primer pair DMC1-P5. Data are presented as the mean values ± SD, *p* values were calculated using a two-tailed Student's t-test. **(d)** Specificity examination of anti-DMC1 antibody. The anti-DMC1 antibody specifically recognized recombinant SUMO-HIS-DMC1 heterologously expressed in *E.coli* rather than SUMO-HIS-RAD51. This experiment was repeated twice independently with similar results. **(e-f)** Validation of DMC1's mono-ubiquitination. Western blot shows the (e) DMC1-GFP or (f) DMC1-FLAG expressed in *E.coli*, tobacco and *Arabidopsis* examined with anti-GFP or -FLAG and -UBQ11 antibodies. Only expression of DMC1-GFP or DMC1-FLAG *in planta* was detected by anti-UBQ11 antibody, but not in *E.coli*. **(g)** Western blot used anti-DMC1 antibody showed that the DMC1 expressed in *E.coli* exhibits the same molecular weight compared with the endogenous DMC1 in Col-0 central inflorescences. Peripheral inflorescences and leaves of Col-0, and central inflorescences of *dmc1-2* were used as negative controls. Tubulin was included as an internal control. Each experiment was repeated three times independently with similar results. Source data are provided as a Source Data file.

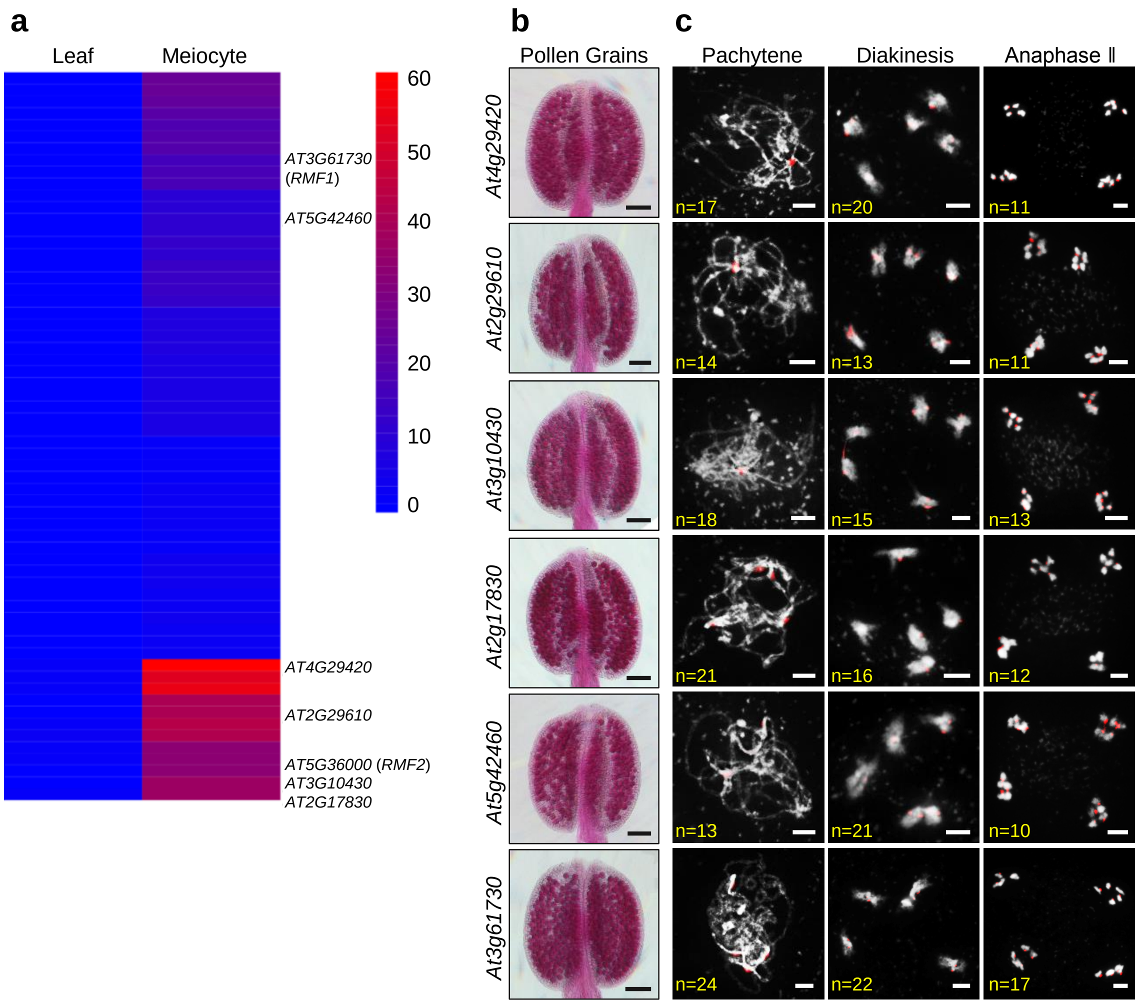

Supplementary Fig. 2. Screening for E3 ubiquitin ligases responsible for DMC1 ubiquitination.

(a) The heat map shows the relative expression level of 62 meiocyte specific or preferential expressed *Arabidopsis* F-box genes. The color legend indicates RPKM in meiocyte and leaf of Col-0. **(b)** Pollen grains stained with Alexander Red from the mutants with mutating individual F-box genes. Each single mutant has normal pollen grain viability similar to Col-0. Bar = 100 μm. For pollen grain viability analysis, 19 anthers for *At4g29420*, 21 anthers for *At2g29610*, 15 anthers for *At3g10430*, 14 anthers for *At2g17830*, 20 anthers for *At5g42460*, 17 anthers for *At3g61730* isolated from more than three independent plants were observed. **(c)** Meiotic phenotype of the mutants as described in (b) assayed by centromere FISH. Each single mutant has normal meiotic chromosome morphology similar to Col-0. Bar = 5 μm. For each meiotic stage in above-mentioned plants, cells isolated from more than three independent plants were observed with similar meiotic chromosome phenotypes. The number of cells observed was labeled in the figures.

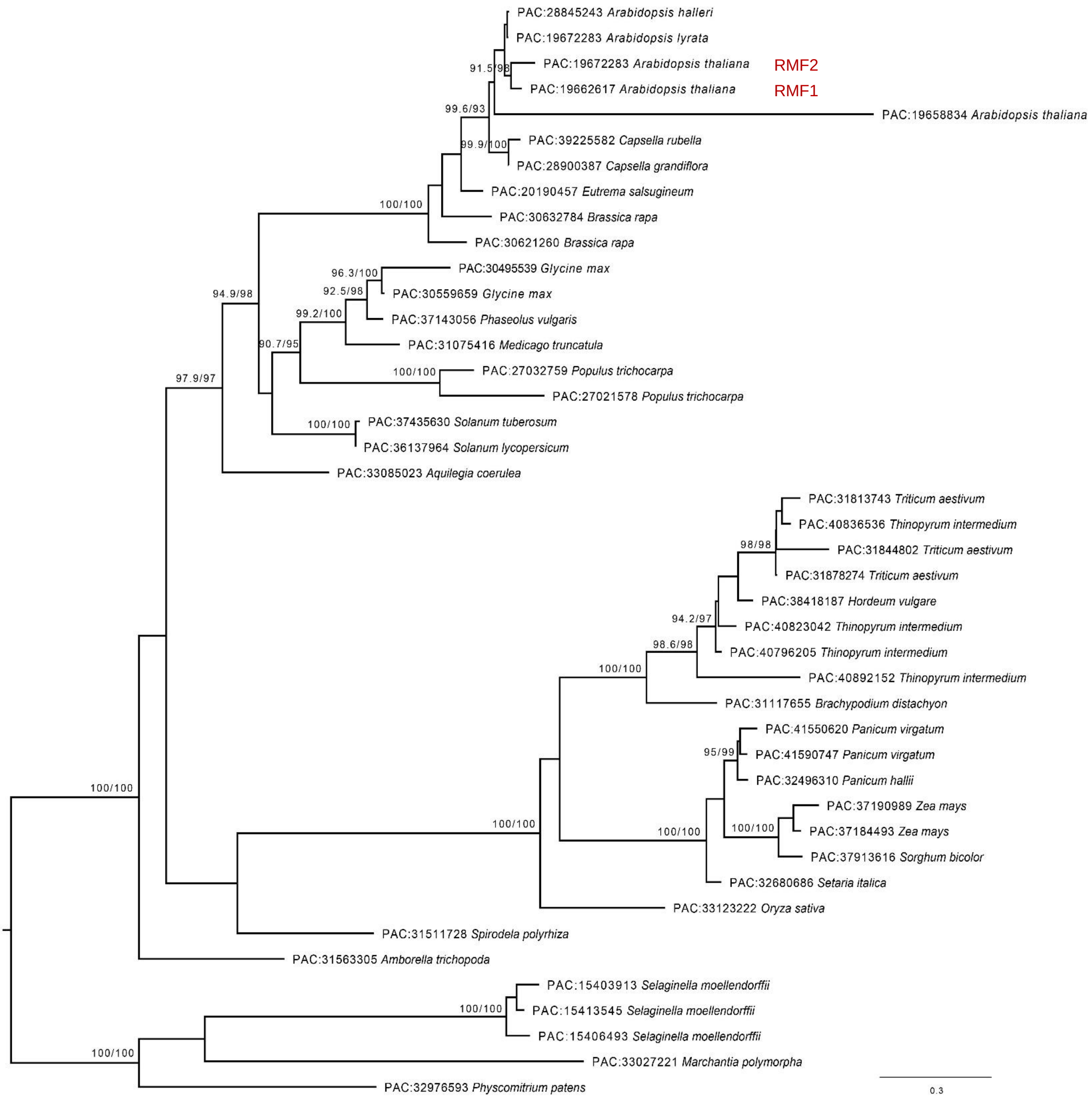

Supplementary Fig. 3. Phylogenetic tree of RMF1/2 and their homologous in plants.

Phylogenetic tree based on the full-length protein sequence of RMF1 and RMF2 and their homologous in land plants. RMF1 and RMF2 are labeled in red. The “SH-aLRT support (%) / ultrafast bootstrap support (%)” are labeled next to the nodes. The branches represent the amino acid substitution rate. The branch tips are labeled with gene accessions in Phytozome v13.

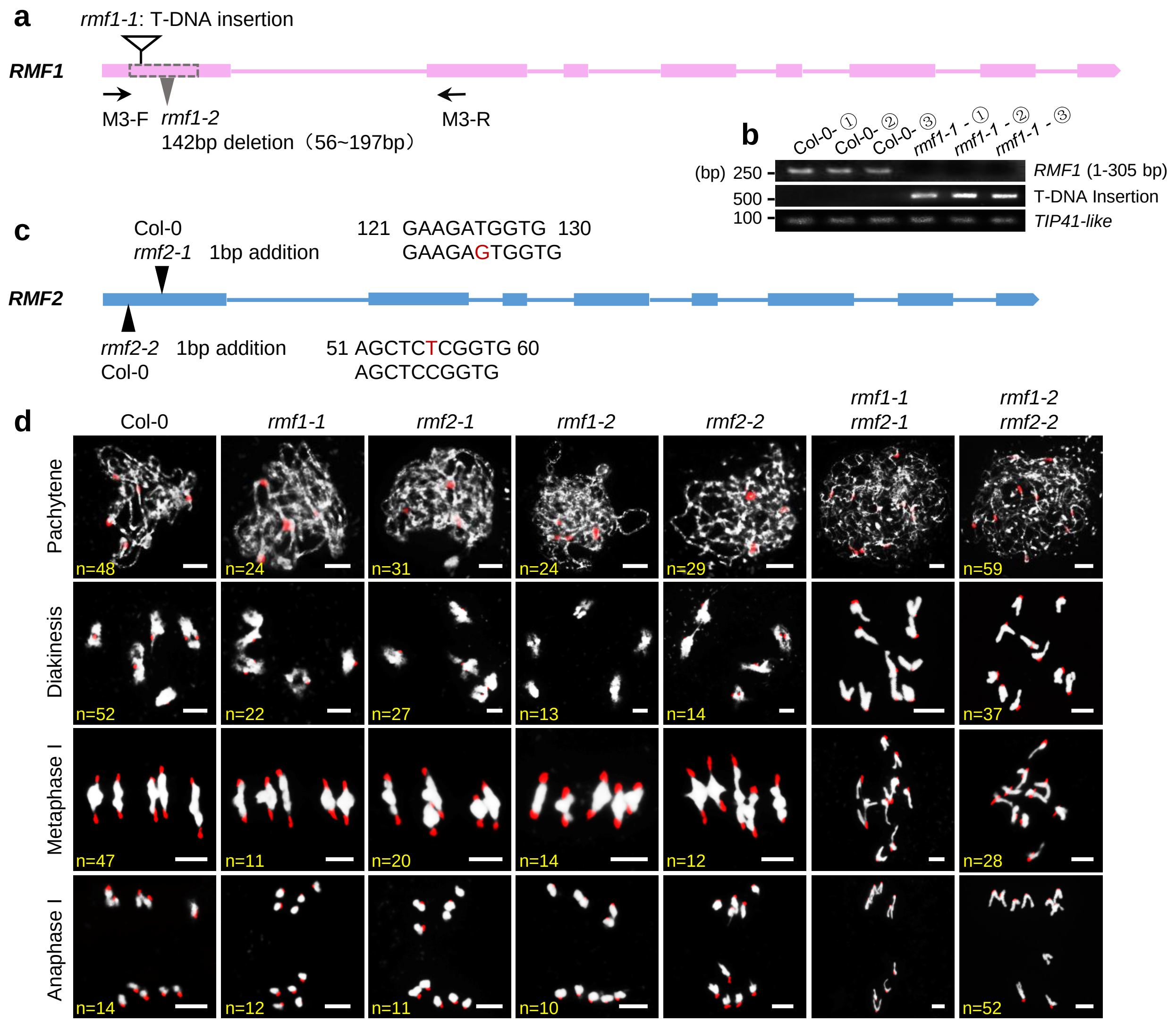

Supplementary Fig. 4. Identification of *rmf1* and *rmf2*.

(a) Schematic diagram of *RMF1* gene structure. Mutant alleles are marked above or below the gene structure with hollow triangle and vertical line (*rmf1-1*, T-DNA insertion) or gray triangle (*rmf1-2*, 142 bp deletion, from 56 to 197 bp). **(b)** Nucleic acid electrophoresis of PCR products amplified by M3-F/M3-R (1-305 bp of *RMF1*-CDS) and LB3/M3-R primers show the *RMF1* CDS bands and T-DNA insertional bands in Col-0 and *rmf1-1 rmf2-1* of three replicates with individual plants. **(c)** Schematic diagram of *RMF2* gene structure. Mutant alleles are marked above (*rmf2-1*, a “G” addition) or below (*rmf2-2*, a “T” addition) the gene structure with black triangles. **(d)** Chromosome morphology at pachytene, diakinesis, metaphase I, anaphase I in Col-0, *rmf1-1*, *rmf2-1*, *rmf1-2*, *rmf2-2*, *rmf1-1 rmf2-1* and *rmf1-2 rmf2-2* assayed by centromere FISH. The single mutants of *rmf1-1*, *rmf2-1*, *rmf1-2*, *rmf2-2* exhibit indistinguishable meiotic phenotype compared with Col-0, while *rmf1-1 rmf2-1* and *rmf1-2 rmf2-2* double mutants are defective in homologous chromosome pair and synapsis with ten univalents at diakinesis. Bar = 5 μm. For each meiotic stage in above-mentioned plants, cells isolated from more than three independent plants were observed with similar meiotic chromosome phenotypes. The number of cells observed was labeled in the figures. Source data are provided as a Source Data file.

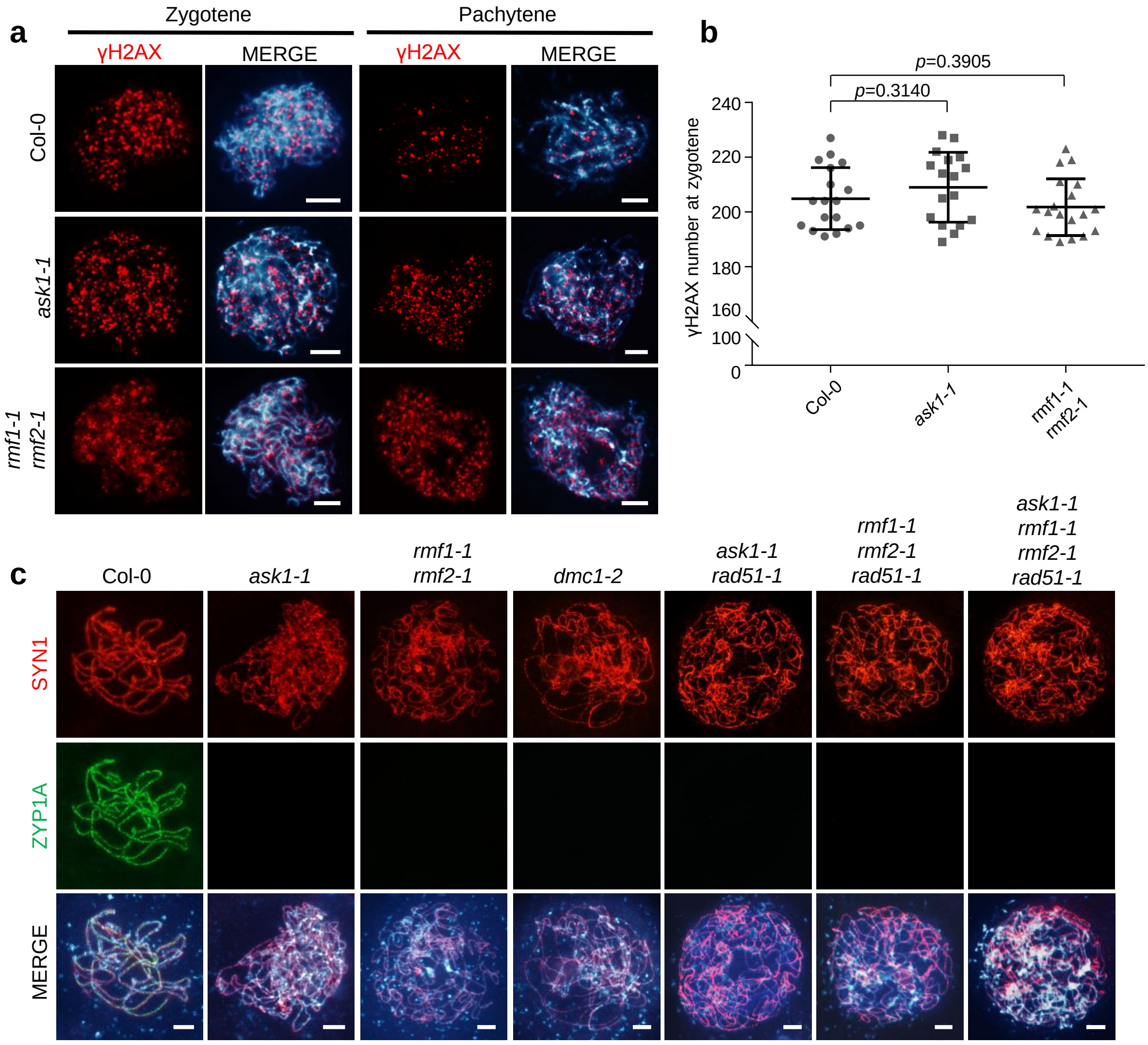

Supplementary Fig. 5. Examination of γH2AX, SYN1 and ZYP1A.

(a) γH2AX localization of Col-0, *ask1-1* and *rmf1-1 rmf2-1* at zygotene and pachytene. Bar = 5 µm. For γH2AX foci number analysis, 18 cells for Col-0 zygotene, 24 cells for Col-0 pachytene, 17 cells for *ask1-1* zygotene, 21 cells for *ask1-1* pachytene, 19 cells for *rmf1-1 rmf2-1* zygotene, 20 cells for *rmf1-1 rmf2-1* pachytene isolated from more than three independent plants were observed. **(b)** Scatter plot of γH2AX foci number of Col-0, *ask1* and *rmf1-1 rmf2-1* at zygotene. Data are presented as the mean values ± SD, *p* values were calculated using a two-tailed Student's t-test. For analyzing γH2AX foci number at zygotene, 18 cells for Col-0, 17 cells for *ask1-1*, 19 cells for *rmf1-1 rmf2-1* isolated from more than three independent plants were used. **(c)** Distribution of SYN1 (red) and ZYP1A (green) in Col-0, *ask1-1*, *rmf1-1 rmf2-1*, *dmc1-2* and *ask1-1 rad51-1*, *rmf1-1 rmf2-1 rad51-1*, *ask1-1 rmf1-1 rmf2-1 rad51-1* at pachytene-like chromosomes compared with Col-0. Bar = 5 µm. For analyzing SYN1 (red) and ZYP1A signal at pachytene or pachytene-like stage, 55 cells for Col-0, 26 cells for *ask1-1*, 30 cells for *rmf1-1 rmf2-1*, 24 cells *dmc1-2*, 14 cells for *ask1-1 rad51-1*, 19 cells for *rmf1-1 rmf2-1 rad51-1*, 16 cells for *ask1-1 rmf1-1 rmf2-1 rad51-1* isolated from more than three independent plants were observed. Source data are provided as a Source Data file.

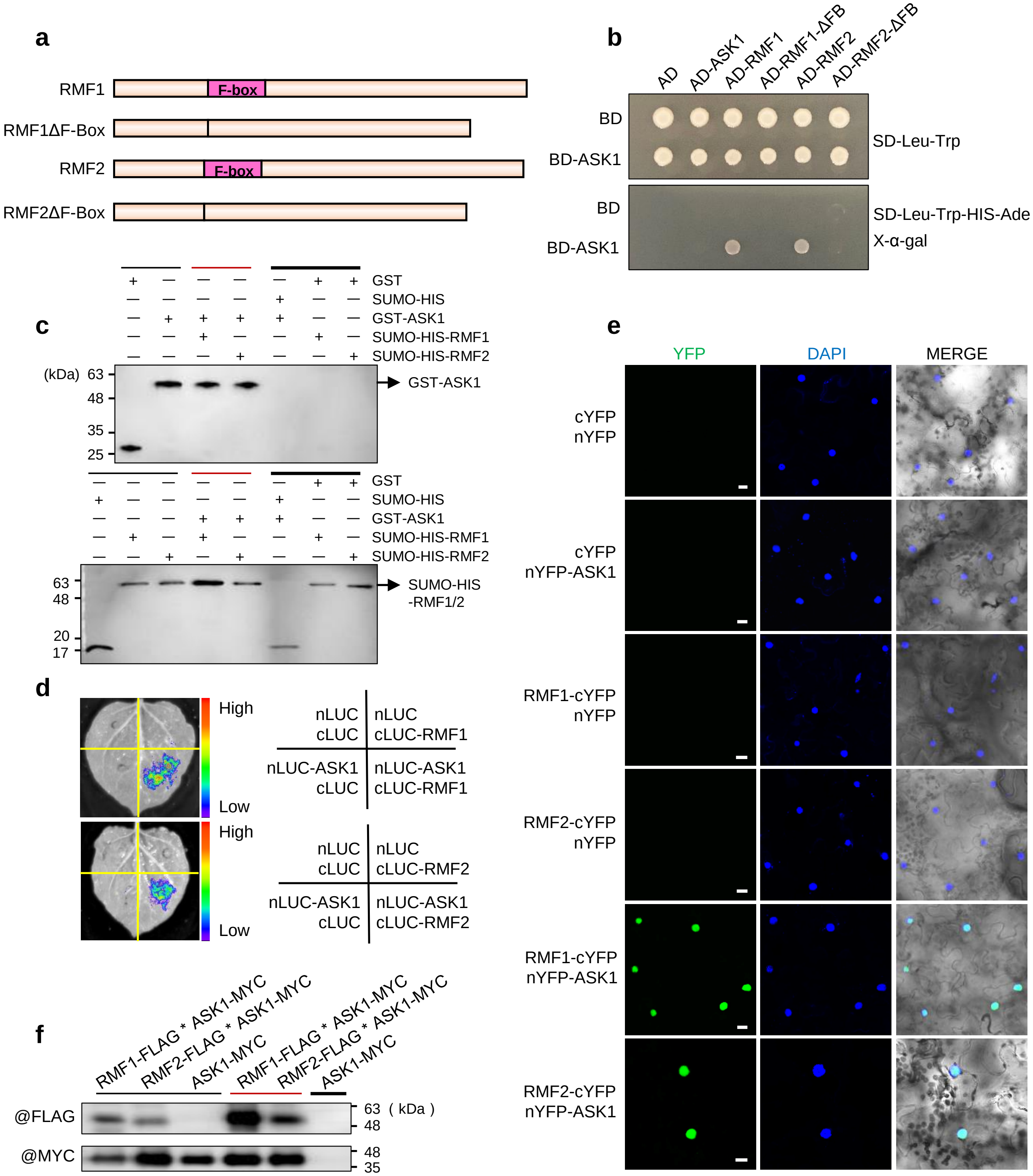

a
RMF1
F-box
RMF1ΔF-Box
RMF2
F-box
RMF2ΔF-Box
b
AD
AD-ASK1
AD-RMF1
AD-RMF1-ΔFB
AD-RMF2
AD-RMF2-ΔFB
BD
BD-ASK1
SD-Leu-Trp
BD
BD-ASK1
SD-Leu-Trp-HIS-Ade
X-α-gal
c
GST
SUMO-HIS
GST-ASK1
SUMO-HIS-RMF1
SUMO-HIS-RMF2
(kDa) 63
48
35
25
GST-ASK1
GST
SUMO-HIS
GST-ASK1
SUMO-HIS-RMF1
SUMO-HIS-RMF2
63
48
20
17
SUMO-HIS
-RMF1/2
d
High
Low
nLUC
cLUC
nLUC
cLUC-RMF1
nLUC-ASK1
cLUC
nLUC-ASK1
cLUC-RMF1
High
Low
nLUC
cLUC
nLUC
cLUC-RMF2
nLUC-ASK1
cLUC
nLUC-ASK1
cLUC-RMF2
e
YFP
DAPI
MERGE
cYFP
nYFP
cYFP
nYFP-ASK1
RMF1-cYFP
nYFP
RMF2-cYFP
nYFP
RMF1-cYFP
nYFP-ASK1
RMF2-cYFP
nYFP-ASK1
f
RMF1-FLAG * ASK1-MYC
RMF2-FLAG * ASK1-MYC
ASK1-MYC
RMF1-FLAG * ASK1-MYC
RMF2-FLAG * ASK1-MYC
ASK1-MYC
@FLAG
@MYC
63 (kDa)
48
48
35

Supplementary Fig. 6. RMF1 and RMF2 interact with ASK1 *in vitro* and *in vivo*.

(a) Schematic diagrams of RMF1 and RMF2 protein structures. The pink rectangles indicate the F-box domain. **(b)** Yeast two-hybrid of the interaction of RMF1/2 with ASK1. Interactions of the corresponding combinations were selected by SD-Leu-Trp-His-Ade X-α-gal media. **(c)** Pull-down assay to test the interactions between RMF1/2 with ASK1. The recombinant GST-ASK1 and SUMO-HIS-RMF1/2 were heterologously expressed in *E.coli*. GST-ASK1 with SUMO-HIS-RMF1 or SUMO-HIS-RMF2 were pulled-down using HIS Resin and examined with anti-GST and -HIS antibodies. Black lines indicate input (thin line) and negative control (thick line), and red line indicates the experimental group. This experiment was repeated three times independently with similar results. **(d)** SLC was used to examine the interactions between RMF1/2 with ASK1. ASK1 was fused to an N-terminal fragment of luciferase (nLUC) and RMF1/2 were fused to a C-terminal fragment of luciferase (cLUC). Paired proteins were infiltrated into tobacco leaves with nLUC or cLUC as negative control. The right panel displays each combination. Robust LUC activities were observed in areas co-infiltrated with nLUC-ASK1/cLUC-RMF1 and nLUC-ASK1/cLUC-RMF2. **(e)** BiFC assay in tobacco showed nuclear signals for RMF1/2-ASK1 interactions. RMF1/2 were fused to a C-terminal fragment of YFP (cYFP) and ASK1 was fused to an N-terminal fragment of YFP (nYFP). Paired proteins were infiltrated into tobacco leaves with nYFP or cYFP as negative control. Nuclear signals were observed in tobacco cells co-infiltrated with RMF1-cYFP/nYFP -ASK1 and RMF2-cYFP/nYFP-ASK1. Bar = 20 μm. This experiment was repeated three times independently with similar results and 30 tobacco nuclei were observed with nuclear signals for each combination. **(f)** Validation of the RMF1-ASK1 and RMF2-ASK1 interaction by transient expression and co-IP in tobacco. Anti-FLAG and -MYC antibodies were used to detect the precipitates immunoprecipitated by anti-FLAG magnetic beads from tobacco leaves co-infiltrated with RMF1-FLAG/ASK1-MYC and RMF2-FLAG/ASK1-MYC. ASK1-MYC was included as a control. Black lines indicate input (thin line) and negative control (thick line), and red line indicates the experimental group. This experiment was repeated three times independently with similar results. Source data are provided as a Source Data file.

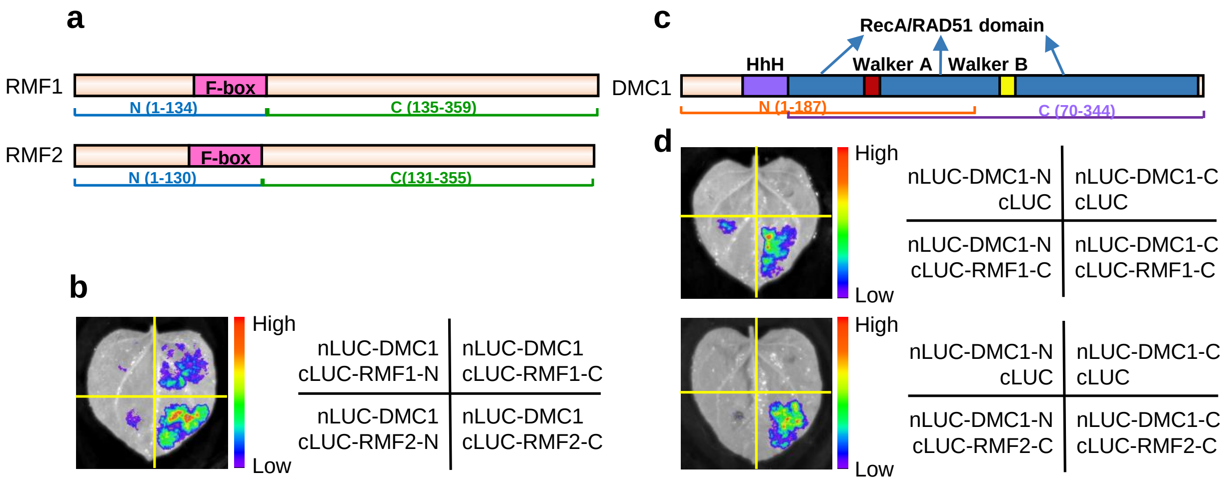

Supplementary Fig. 7. C-terminus of RMF1/2 (RMF1/2-C) interact with C-terminus of DMC1 (DMC1-C).

(a) Schematic diagram of RMF1 and RMF2 protein structures. RMF1-N and -C contain 1-134 and 135-359 amino acids (AAs), respectively, and are designated RMF2-N (1-130 AAs) and RMF2-C (131-355 AAs). The pink rectangles represents the F-box domain. **(b)** SLC was used to test the interactions between N- and C-terminal of RMF1 (RMF1-N, RMF1-C) and RMF2 (RMF2-N, RMF2-C) with DMC1. DMC1 was fused to an N-terminal fragment of luciferase (nLUC), while RMF1-N, RMF1-C and RMF2-N, RMF2-C were fused to a C-terminal fragment of luciferase (cLUC). The right panel displays each combination. Robust LUC activities were observed in areas co-infiltrated with nLUC-DMC1/cLUC-RMF1-C and nLUC-DMC1/cLUC-RMF2-C. **(c)** Schematic diagram of DMC1 protein structure. DMC1-N (1-187 AAs), DMC1-C (70-344 AAs), respectively. The purple rectangle represents HhH (helix-hairpin-helix) nonspecific DNA-binding motif, while the red and yellow rectangles represent Walker A and Walker B ATPase motifs, and the blue rectangles represents RecA/RAD51 domain. **(d)** SLC used to test the interactions between RMF1-C, RMF2-C with DMC1-N and DMC1-C. DMC1-N and DMC1-C were fused to an N-terminal fragment of luciferase (nLUC), while RMF1-C and RMF2-C were fused to a C-terminal fragment of luciferase (cLUC). The right panel displays each combination. Robust LUC activities were observed in areas co-infiltrated with nLUC-DMC1-C/cLUC-RMF1-C and nLUC-DMC1-C/cLUC-RMF2-C.

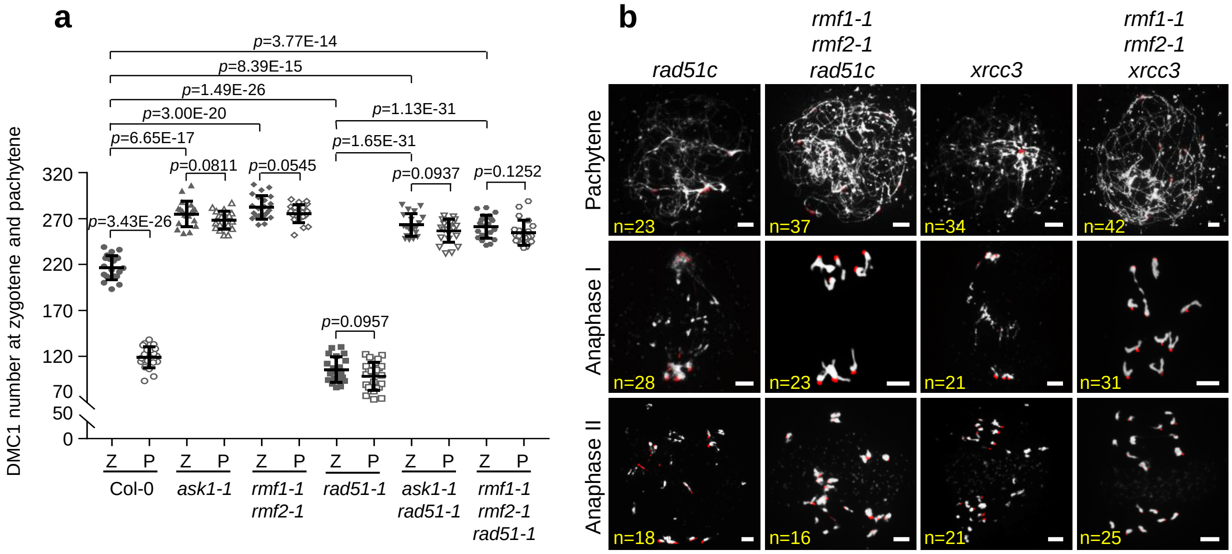

Supplementary Fig. 8. DMC1 can substitute RAD51 in the absence of ASK1 and/or RMF1/2.

(a) Scatter plot of DMC1 foci number of Col-0, *ask1-1*, *rmf1-1 rmf2-1* and *rad51-1*, *ask1-1 rad51-1*, *rmf1-1 rmf2-1 rad51-1* at zygotene (Z) and pachytene (P). Data are presented as the mean values ± SD, *p* values were calculated using a two-tailed Student's t-test. For analyzing DMC1 foci number at zygotene and pachytene, 22 cells for Col-0 zygotene, 22 cells for Col-0 pachytene, 20 cells for *ask1-1* zygotene, 20 cells for *ask1-1* pachytene, 22 cells for *rmf1-1 rmf2-1* zygotene, 21 cells for *rmf1-1 rmf2-1* pachytene, 20 cells for *rad51-1* zygotene, 25 cells for *rad51-1* pachytene, 20 cells for *ask1-1 rad51-1* zygotene, 21 cells for *ask1-1 rad51-1* pachytene, 21 cells for *rmf1-1 rmf2-1 rad51-1* zygotene, 21 cells for *rmf1-1 rmf2-1 rad51-1* pachytene isolated from more than 3 independent plants were used. **(b)** Chromosome morphology of *rad51c*, *rmf1-1 rmf2-1 rad51c* and *xrcc3*, *rmf1-1 rmf2-1 xrcc3* at pachytene, anaphase I and anaphase II assayed by centromere FISH. The *rad51c* and *xrcc3* single mutants have severe chromosomes entanglements and fragmentation, which are dramatically decreased in the *rmf1-1 rmf2-1 rad51c* and *rmf1-1 rmf2-1 xrcc3* higher-order mutants. Bar = 5 µm. For each meiotic stage in above-mentioned plants, cells isolated from more than three independent plants were observed with similar meiotic chromosome phenotypes. The number of cells observed was labeled in the figures. Source data are provided as a Source Data file.

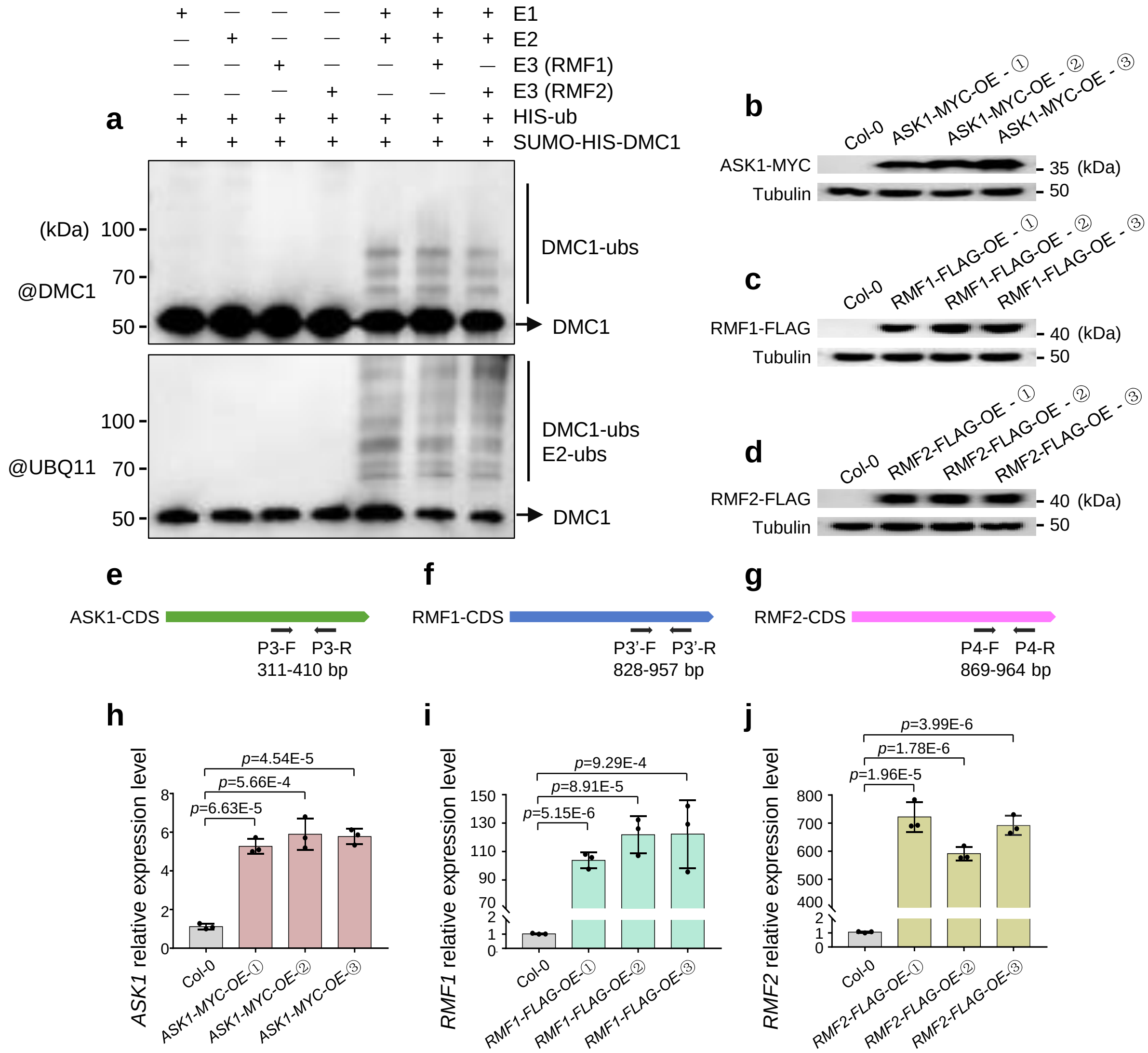
a
E1
E2
E3 (RMF1)
E3 (RMF2)
HIS-ub
SUMO-HIS-DMC1
(kDa) 100
70
50
@DMC1
DMC1-ubs
DMC1
@UBQ11
DMC1-ubs
E2-ubs
b
Col-0
ASK1-MYC-OE - ①
ASK1-MYC-OE - ②
ASK1-MYC-OE - ③
ASK1-MYC
Tubulin
35 (kDa)
c
RMF1-FLAG-OE - ①
RMF1-FLAG-OE - ②
RMF1-FLAG-OE - ③
RMF1-FLAG
40 (kDa)
d
RMF2-FLAG-OE - ①
RMF2-FLAG-OE - ②
RMF2-FLAG-OE - ③
RMF2-FLAG
e
ASK1-CDS
P3-F P3-R
311-410 bp
f
RMF1-CDS
P3'-F P3'-R
828-957 bp
g
RMF2-CDS
P4-F P4-R
869-964 bp
h
ASK1 relative expression level
p=4.54E-5
p=5.66E-4
p=6.63E-5
i
RMF1 relative expression level
p=9.29E-4
p=8.91E-5
p=5.15E-6
j
RMF2 relative expression level
p=3.99E-6
p=1.78E-6
p=1.96E-5

Supplementary Fig. 9. DMC1 can be ubiquitinated *in vitro* and its relative expression level in the *ASK1, RMF1, RMF2* overexpression (OE) plants.

(a) Ubiquitination of DMC1 *in vitro*. The recombinant SUMO-HIS-DMC1 were heterologously expressed in *E.coli*. Anti-FLAG immunoprecipitates from central inflorescences of *RMF1/2-FLAG* transgenic plants (validated in Supplementary Fig. 8c, d and i, j) were used as E3 and recombinant SUMO-HIS-DMC1 as substrate. Different combinations of E1, E2, E3, substrate and ubiquitin (ub) are shown. The proteins were detected by western blot analysis using anti-DMC1 and -UBQ11 antibodies. The ladder above the main DMC1 band indicates the ubiquitinated DMC1 (with anti-DMC1 antibody) or ubiquitinated DMC1 and E2 (with anti-UBQ11 antibody). This experiment was repeated three times independently with similar results. **(b-d)** Western blot shows the expression of ASK1-MYC, RMF1-FLAG and RMF2-FLAG in inflorescences of three individual plants from the (b) *ASK1-MYC-OE*, (c) *RMF1-FLAG-OE*, (d) *RMF2-FLAG-OE* transgenic line used in Fig. 6c (b, c, d) and Supplementary Fig. 8a (c, d), respectively. The *ASK1/RMF1/RMF2* CDS sequences (without stop codon) with MYC or FLAG were cloned and placed under the control of the *ACT7* promoter. **(e-g)** Schematic diagram of *ASK1*, *RMF1* and *RMF2* CDS to show the primer pair used for qRT-PCR of (e) *ASK1-MYC-OE* (ASK1-P3), (f) *RMF1-FLAG-OE* (RMF1-P3’), (g) *RMF2-FLAG-OE* (RMF2-P4) transgenic plants marked below the gene structure as black arrows. **(h-j)** qRT-PCR shows the relative expression level of the examined genes in inflorescences of Col-0 and three biologically independent plants from the (h) *ASK1-MYC-OE*, (i) *RMF1-FLAG-OE*, (j) *RMF2-FLAG-OE* transgenic line used in Fig. 6c (h, i, j) and Supplementary Fig. 8a (i, j) with the primer pair ASK1-P3, RMF1-P3’, RMF2-P4, respectively. Data are presented as the mean values $\pm$ SD, p values were calculated using a two-tailed Student’s t-test. Source data are provided as a Source Data file.

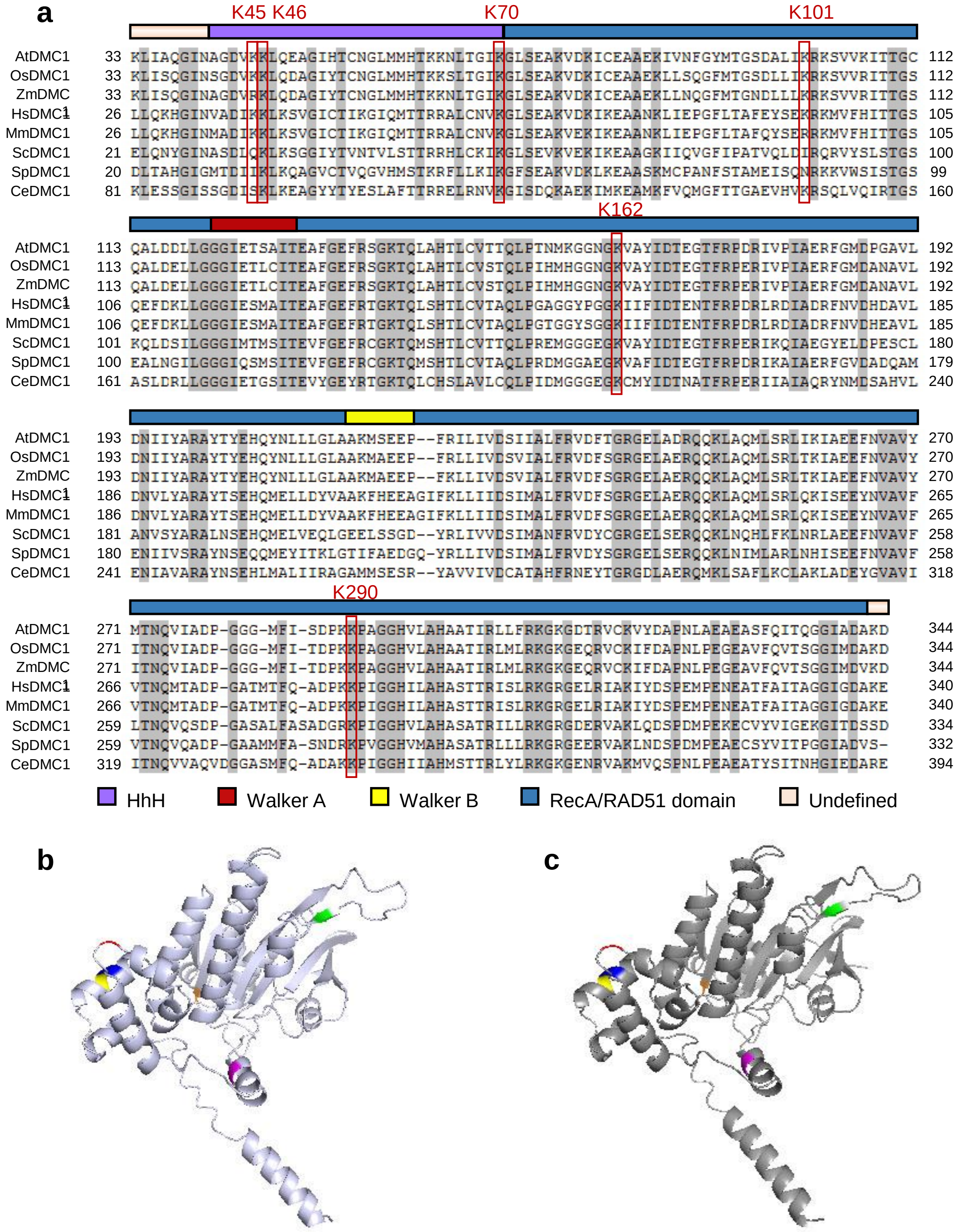

Supplementary Fig. 10. Alinement of DMC1 amino acid sequences and functional characterization of DMC1 ubiquitination sites.

(a) Alignment of DMC1 amino acid sequences in *Arabidopsis* (AtDMC1), rice (OsDMC1), maize (ZmDMC1), human (HsDMC1), mouse (MmDMC1), budding yeast (ScDMC1), fission yeast (SpDMC1), and *C.elegant* (CeDMC1). The six potential ubiquitination sites of DMC1 (K45, K46, K70, K101, K162, and K290) are marked by red rectangles. The schematic diagram of DMC1 protein structure is labeled above the alignment. **(b-c)** The ribbon diagram of predicted (b) DMC1 and (c) DMC1-6KR protein structures using the AlphaFold protein structure database and AlphaFold2 protein structure prediction database. The six potential ubiquitination sites (lysines) of DMC1 or six lysines mutated to arginines within DMC1 are colored in yellow (K/R45), blue (K/R46), red (K/R70), magenta (K/R101), orange (K/R162), and green (K/R290), respectively.

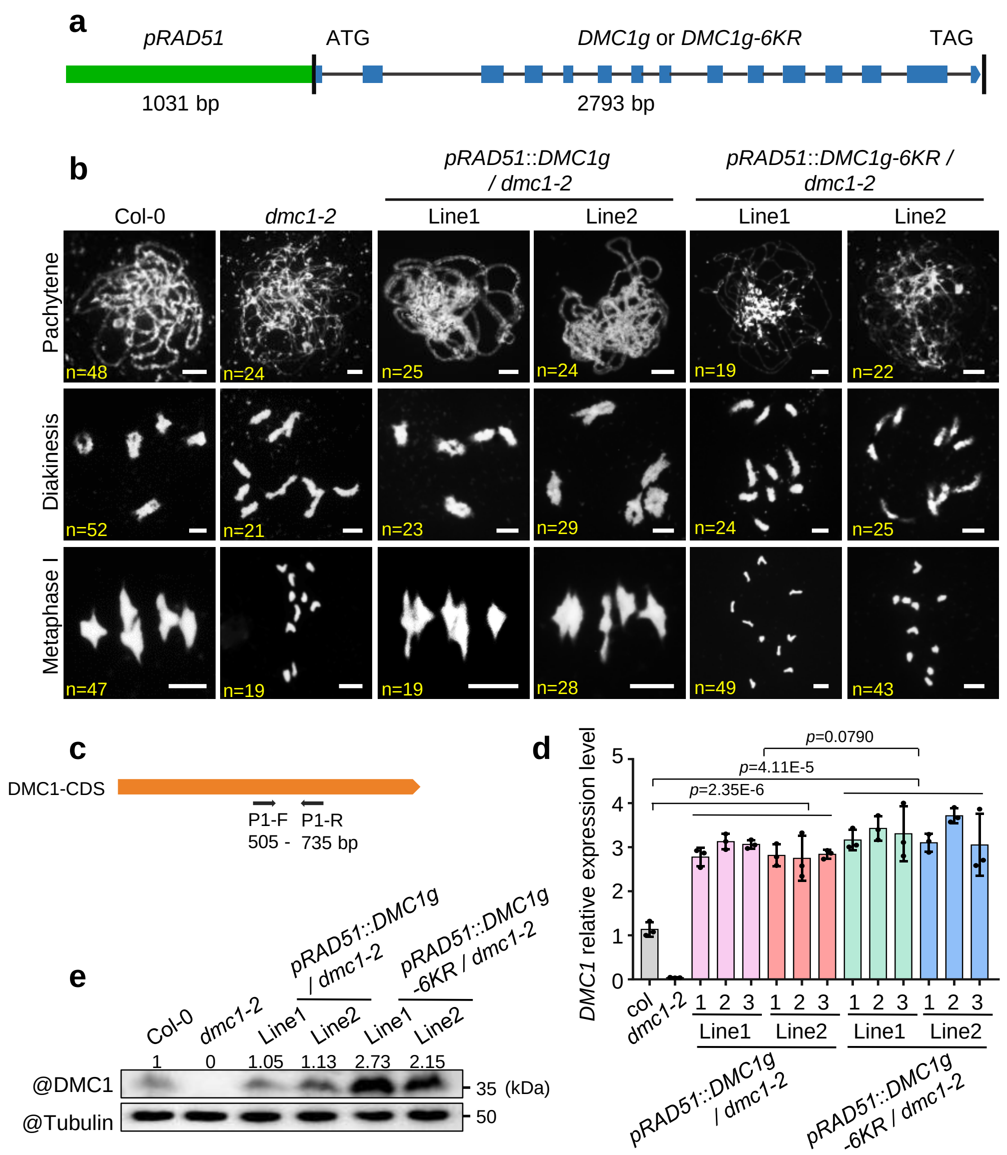
a
pRAD51
ATG
DMC1g or DMC1g-6KR
TAG
1031 bp
2793 bp
b
pRAD51::DMC1g / dmc1-2
pRAD51::DMC1g-6KR / dmc1-2
Col-0
dmc1-2
Line1
Line2
Line1
Line2
Pachytene
Diakinesis
Metaphase I
n=48
n=24
n=25
n=24
n=19
n=22
n=52
n=21
n=23
n=29
n=24
n=25
n=47
n=19
n=19
n=28
n=49
n=43
c
DMC1-CDS
P1-F
P1-R
505 -
735 bp
d
DMC1 relative expression level
p=0.0790
p=4.11E-5
p=2.35E-6
col
dmc1-2
Line1
Line2
Line1
Line2
pRAD51::DMC1g / dmc1-2
pRAD51::DMC1g -6KR / dmc1-2
e
Col-0
dmc1-2
Line1
Line2
Line1
Line2
pRAD51::DMC1g / dmc1-2
pRAD51::DMC1g -6KR / dmc1-2
1
0
1.05
1.13
2.73
2.15
@DMC1
@Tubulin
35 (kDa)
50

Supplementary Fig. 11. Functional characterization of DMC1 ubiquitination sites.

(a) Schematic diagram of pRAD51::DMC1g or pRAD51::DMC1g-6KR construct using *DMC1* or *DMC1-6KR* genomic sequences (ATG to stop codon, indicated as *DMC1g* or *DMC1g-6KR*) driven under the control of the *RAD51* promoter. **(b)** Chromosome morphology of Col-0, *dmc1-2*, and two independent lines of *pRAD51*::*DMC1g/dmc1-2* and *pRAD51*::*DMC1g-6KR/dmc1-2*, respectively. The meiotic defects are restored in *pRAD51*::*DMC1g/dmc1-2* but not in *pRAD51*:: *DMC1g-6KR/dmc1-2*. Bar = 5 μm. For each meiotic stage in above-mentioned plants, cells isolated from more than three independent plants were observed with similar meiotic chromosome phenotypes. The number of cells observed was labeled in the figures. **(c)** Schematic diagram of *DMC1* CDS to show the primer pair used for qRT-PCR in (d) (DMC1-P1). **(d)** qRT-PCR shows the relative expression level of *DMC1* in inflorescences of Col-0, *dmc1-2*, and three biologically independent plants from two independent lines of *pRAD51*::*DMC1g /dmc1-2* and *pRAD51*::*DMC1g-6KR/dmc1-2*. Data are presented as the mean values ± SD, *p* values between different groups (Col-0 with two independent lines of *pRAD51*::*DMC1g/dmc1-2*, Col-0 with two independent lines of *pRAD51*:: *DMC1g-6KR/dmc1-2*, and two independent lines of *pRAD51*::*DMC1g/dmc1-2* with two independent lines of *pRAD51*::*DMC1g-6KR/dmc1-2*) were calculated using a one-way ANOVA test. **(e)** The protein level of DMC1 is slightly higher in *pRAD51*::*DMC1g/dmc1-2*, and significantly higher in *pRAD51*:: *DMC1g-6KR/ dmc1-2* compared with Col-0. Anti-DMC1 antibody was used to examine the protein level of DMC1 in central inflorescences of Col-0, *dmc1-2*, two independent lines of *pRAD51*::*DMC1g/dmc1-2* and *pRAD51*:: *DMC1g-6KR /dmc1-2* plants, respectively. Tubulin was included as an internal control. Source data are provided as a Source Data file.

Supplementary Table 1. Five lysine residues identified by mass spectrometry as potential ubiquitination sites of DMC1.

Sequence	# Proteins	# Protein Groups	Protein Accessions	Modifications	Area	IonScore	Exp Value
LIAQGINAGDVkK	1	1	AT3G22880.1	K12(GG) K45	5.621E6	36	0.004420923
KNLTGIkGLSEAK	1	1	AT3G22880.1	K7(GG) K70	1.143E7	33	0.011289984
IVNFGYMTGSDALIkR	1	1	AT3G22880.1	K15(GG) K101	1.594E6	44	0.001380527
GGNGkVAYIDTEGTFRPDR	1	1	AT3G22880.1	K5(GG) K162	1.367E6	31	0.03294062
kPAGGHVLAHAATIR	1	1	AT3G22880.1	K1(GG) K290	1.368E7	56	5.24422E-05
