# Supplementary material for: SCFRMF mediates degradation of the meiosis-specific recombinase DMC1
Source: Nat Commun. 2023 Aug 19;14:5044. doi: 10.1038/s41467-023-40799-5 (PMC10439943; doi:10.1038/s41467-023-40799-5)
Supplement: Supplementary file 3 — Description of Additional Supplementary Files [file 41467_2023_40799_MOESM3_ESM.pdf]

Description of Additional Supplementary Files

File Name: Supplementary Data 1
Description: The proteins identified by IP-MS analysis of DMC1.

File Name: Supplementary Data 2
Description: The proteins identified by IP-MS analysis of DMC1 with background removing.

File Name: Supplementary Data 3
Description: The relative expression level of 62 *Arabidopsis* meiotic preferentially expressed genes encoding F-box proteins in meiocyte and leaf.

File Name: Supplementary Data 4
Description: List of primers used in this study.
