# Supplementary material for: SCFRMF mediates degradation of the meiosis-specific recombinase DMC1
Source: Nat Commun. 2023 Aug 19;14:5044. doi: 10.1038/s41467-023-40799-5 (PMC10439943; doi:10.1038/s41467-023-40799-5)
Supplement: Supplementary file 8 — Reporting Summary [file 41467_2023_40799_MOESM8_ESM.pdf]

nature portfolio

Corresponding author(s): Yingxiang Wang

Last updated by author(s): Jul 29, 2023

Reporting Summary

Nature Portfolio wishes to improve the reproducibility of the work that we publish. This form provides structure for consistency and transparency in reporting. For further information on Nature Portfolio policies, see our Editorial Policies and the Editorial Policy Checklist.

Statistics

For all statistical analyses, confirm that the following items are present in the figure legend, table legend, main text, or Methods section.

n/a	Confirmed	
☐	☒	The exact sample size (*n*) for each experimental group/condition, given as a discrete number and unit of measurement
☐	☒	A statement on whether measurements were taken from distinct samples or whether the same sample was measured repeatedly
☐	☒	The statistical test(s) used AND whether they are one- or two-sided<br>*Only common tests should be described solely by name; describe more complex techniques in the Methods section.*
☐	☒	A description of all covariates tested
☐	☒	A description of any assumptions or corrections, such as tests of normality and adjustment for multiple comparisons
☐	☒	A full description of the statistical parameters including central tendency (e.g. means) or other basic estimates (e.g. regression coefficient) AND variation (e.g. standard deviation) or associated estimates of uncertainty (e.g. confidence intervals)
☐	☒	For null hypothesis testing, the test statistic (e.g. *F*, *t*, *r*) with confidence intervals, effect sizes, degrees of freedom and *P* value noted<br>*Give P values as exact values whenever suitable.*
☒	☐	For Bayesian analysis, information on the choice of priors and Markov chain Monte Carlo settings
☒	☐	For hierarchical and complex designs, identification of the appropriate level for tests and full reporting of outcomes
☒	☐	Estimates of effect sizes (e.g. Cohen's *d*, Pearson's *r*), indicating how they were calculated

Our web collection on statistics for biologists contains articles on many of the points above.

Software and code

Policy information about availability of computer code

Data collection	The plants and stems were photographed using a Canon digital camera SX20 IS (Canon). Images of stained pollen and microspores were obtained using a Zeiss Axio Scope A1 microscope (Zeiss). Image of chromosome spreading, fluorescence in situ hybridization (FISH) with a centromere probe, and immunofluorescence experiment were obtained using a Zeiss Axio Scope A1 microscope (Zeiss). Image of Split luciferase complementation imaging assay were obtained using a living plant imaging system NightSHADE LB 985 (Berthold Technology) with indiGo software (v2.0.5.0). Image of Bimolecular Fluorescent Complimentary (BiFC) assay were obtained using a laser confocal microscope FV3000 (Olympus). Proteome Discoverer (v1.4, Thermo Fisher Scientific) with an in-house Mascot Server (v2.7, Matrix Science) was used for LC-MS raw data analysis.
Data analysis	Excel 2019 (Microsoft);<br>PANTHER database; http://www.pantherdb.org/;<br>Prism 7 (GraphPad); https://www.graphpad.com/;<br>R (v3.4.2); https://cran.r-project.org;<br>Photoshop CS6 (Adobe);<br>ImageJ (v2.1.1); https://imagej.nih.gov/ij/;<br>HMMER (v3.3.2); http://hmmer.org/;<br>Phytozome v13; https://phytozome-next.jgi.doe.gov;<br>IQ-Tree (v2.2.0); https://www.iqtree.org;<br>FigTree (v1.4.4); http://tree.bio.ed.ac.uk/software/figtree;<br>PyMOL (v2.6.0); https://www.lfd.uci.edu/~gohlke/pythonlibs/#pymol-open-source;<br>MEGA (v6.0); https://www.megasoftware.net/;<br>DNAMAN (v6.0.3); https://en.freedownloadmanager.org/Windows-PC/DNAMAN.html;

Proteome Discoverer (v1.4, Thermo Fisher Scientific);
Mascot Server (v2.7, Matrix Science).

For manuscripts utilizing custom algorithms or software that are central to the research but not yet described in published literature, software must be made available to editors and reviewers. We strongly encourage code deposition in a community repository (e.g. GitHub). See the Nature Portfolio guidelines for submitting code & software for further information.

Data

Policy information about availability of data

All manuscripts must include a data availability statement. This statement should provide the following information, where applicable:

- Accession codes, unique identifiers, or web links for publicly available datasets
- A description of any restrictions on data availability
- For clinical datasets or third party data, please ensure that the statement adheres to our policy

The amino acid, CDS and genomic sequences of RMF1 (https://www.arabidopsis.org/servlets/TairObject?id=36024&type=locus), RMF2 (https://www.arabidopsis.org/servlets/TairObject?id=133155&type=locus), ASK1 (https://www.arabidopsis.org/servlets/TairObject?id=137570&type=locus), DMC1 (https://www.arabidopsis.org/servlets/TairObject?id=37395&type=locus), RAD51 (https://www.arabidopsis.org/servlets/TairObject?id=130976&type=locus) are available at TAIR under accession numbers AT3G61730 (RMF1), AT5G36000 (RMF2), AT1G75950 (ASK1), At3G22880 (DMC1), AT5G20850 (RAD51), respectively. Homologs of Arabidopsis RMF1 and RMF2 for phylogenetic analysis were identified using HMMER3 to search against a protein database from selected plant species from Phytozome v13 (https://phytozome-next.jgi.doe.gov). The predicted protein structure of DMC1 and DMC1-6KR were obtained from the online AlphaFold protein structure database (https://alphafold.ebi.ac.uk/) and the online AlphaFold2 protein structure prediction database (https://colab.research.google.com/github/sokrypton/ColabFold/blob/main/AlphaFold2.ipynb).
Data supporting the findings of this work are available within the paper and its Supplementary Information files. The mass spectrometry proteomics data generated in this study have been deposited in the ProteomeXchange under accession code PXD038116 and PXD038126. Source data are provided with this paper.

Human research participants

Policy information about studies involving human research participants and Sex and Gender in Research.

Reporting on sex and gender	N/A
Population characteristics	N/A
Recruitment	N/A
Ethics oversight	N/A

Note that full information on the approval of the study protocol must also be provided in the manuscript.

Field-specific reporting

Please select the one below that is the best fit for your research. If you are not sure, read the appropriate sections before making your selection.

☒ Life sciences ☐ Behavioural & social sciences ☐ Ecological, evolutionary & environmental sciences

For a reference copy of the document with all sections, see nature.com/documents/nr-reporting-summary-flat.pdf

Life sciences study design

All studies must disclose on these points even when the disclosure is negative.

Sample size	Sample size was determined refer to previous studies and sufficient for statistical analyses. Each sample size was stated in the figures, figure legends or main text.
Data exclusions	No data were excluded from the final analyses.
Replication	All analyses in the study were repeated more than 2 times depending on the different purpose for each experiment. All attempts at replication were successful.
Randomization	All candidate samples in the study including plants, bacterial strains, micrographs of cell, nucleus and chromosome were randomly selected. All samples for biochemistry assays were also randomly selected in this study.
Blinding	All data in the study were collected from blinded to group allocation.

Reporting for specific materials, systems and methods

We require information from authors about some types of materials, experimental systems and methods used in many studies. Here, indicate whether each material, system or method listed is relevant to your study. If you are not sure if a list item applies to your research, read the appropriate section before selecting a response.

Materials & experimental systems

n/a	Involved in the study
☐	☒ Antibodies
☒	☐ Eukaryotic cell lines
☒	☐ Palaeontology and archaeology
☒	☐ Animals and other organisms
☒	☐ Clinical data
☒	☐ Dual use research of concern

Methods

n/a	Involved in the study
☒	☐ ChIP-seq
☒	☐ Flow cytometry
☒	☐ MRI-based neuroimaging

Antibodies

Antibodies used

Goat anti-Rat IgG (H+L) Cross-Adsorbed Secondary Antibody, Alexa Fluor™ 488; Invitrogen; Cat. # A-11006; 1:200 dilution;
Goat anti-Rabbit IgG (H+L) Highly Cross-Adsorbed Secondary Antibody, Alexa Fluor™ 555; Invitrogen; Cat. # A-21428; 1:1000 dilution;
MBP-Tag Polyclonal Antibody (Rabbit); Proteintech; Cat. # 15089-1-AP; 1:2000 dilution;
His-Tag Antibody (Mouse); Abmart; Cat. # M30111; Clone No.: 10E2; 1:2000 dilution;
GST-Tag Antibody (Mouse); Abmart; Cat. # M20007; Clone No.: 12G8; 1:2000 dilution;
Anti-FLAG Monoclonal Antibody (Mouse); GNI; Cat. # GNI4110-FG; Clone No.: 1E6; 1:2000 dilution;
Anti-GFP Monoclonal Antibody (Mouse); GNI; Cat. # GNI4110-GP; Clone No.: 8C1; 1:2000 dilution;
Anti-MYC Tag Antibody (Mouse); Sigma-Aldrich; Cat. # 05-724-25UG; Clone No.: 4A6; 1:2000 dilution;
Goat anti-Mouse IgG-HRP Secondary Antibody; GNI; Cat. # GNI9310-M; 1:2000 dilution;
Goat anti-Rabbit IgG-HRP Secondary Antibody; GNI; Cat. # GNI9310-R; 1:2000 dilution;
Anti-UBQ11 Antibody (Rabbit); Agrisera; Cat. # AS08 307A; 1:5000 dilution;
Anti-β-Tubulin Antibody (Mouse); Abmart; Cat. # M20005; Clone No.: C66; 1:2000 dilution;
HSC70 (plant) monoclonal Antibody (Mouse); ENZO; Cat. # ADI-SPA-818-F; Clone No.: 1D9; 1:2000 dilution;
SYN1 Rabbit Polyclonal Antibody was raised in rabbits and generated by Shanghai Ango Biotechnology CO, China, (Wang et al., PLoS Genet, 2020); 1:200 dilution;
ZYP1A Rat Polyclonal Antibody was raised in rats and generated by GL Biochem, China, (Wang et al., PLoS Genet, 2012); 1:200 dilution;
γH2AX Rabbit Polyclonal Antibody was raised in rabbits and generated by ABMART Inc., China, (Wang et al., PLoS Genet, 2020); 1:200 dilution;
DMC1 Rabbit Polyclonal Antibody was raised in rabbits and generated by Shanghai Ango Biotechnology CO, China, (Wang et al., Plant Physiol, 2019); 1:200 dilution for immunofluorescence and 1:500 dilution for western blot.

Validation

The validation statement of commercial antibodies can be found in the manufacturers' websites :
Goat anti-Rat IgG (H+L) Cross-Adsorbed Secondary Antibody, Alexa Fluor™ 488 (https://www.thermofisher.cn/cn/zh/antibody/product/Goat-anti-Rat-IgG-H-L-Cross-Adsorbed-Secondary-Antibody-Polyclonal/A-11006) ;
Goat anti-Rabbit IgG (H+L) Highly Cross-Adsorbed Secondary Antibody, Alexa Fluor™ 555 (https://www.thermofisher.cn/cn/zh/antibody/product/Goat-anti-Rabbit-IgG-H-L-Cross-Adsorbed-Secondary-Antibody-Polyclonal/A-21428);
MBP-Tag Polyclonal Antibody (Rabbit) (https://www.ptgcn.com/products/MBP-Tag-Antibody-15089-1-AP.htm);
His-Tag Antibody (Mouse) (http://www.ab-mart.com.cn/page.aspx?node=%2059%20&id=%20974);
GST-Tag Antibody (Mouse) (http://www.ab-mart.com.cn/page.aspx?node=%2059%20&id=%20967);
Anti-FLAG Monoclonal Antibody (Mouse) (http://www.gnimission.com/resource/article/86);
Anti-GFP Monoclonal Antibody (Mouse) (http://www.gnimission.com/resource/article/88);
Anti-MYC Tag Antibody (Mouse) (https://www.sigmaaldrich.cn/CN/zh/product/mm/05724);
Goat anti-Mouse IgG-HRP Secondary Antibody (http://www.gnimission.com/resource/article/5);
Goat anti-Rabbit IgG-HRP Secondary Antibody (http://www.gnimission.com/resource/article/2);
Anti-UBQ11 Antibody (Rabbit) (https://www.agrisera.com/en/artiklar/ubq11-ubiquitin-.html);
Anti-β-Tubulin Antibody (Mouse) (http://www.ab-mart.com.cn/page.aspx?node=59&id=983);
HSC70 (plant) monoclonal Antibody (https://www.enzolifesciences.com/ADI-SPA-818/hsc70-plant-monoclonal-antibody-1d9/).
Arabidopsis SYN1 Rabbit Polyclonal Antibody (Wang et al., PLoS Genet, 2020), ZYP1A Rat Polyclonal Antibody (Wang et al., PLoS Genet, 2012), and γH2AX Rabbit Polyclonal Antibody (Wang et al.,PLoS Genet, 2020) have been validated previously. Arabidopsis DMC1 Rabbit Polyclonal Antibody has been validated previously in (Wang et al., Plant Physiol, 2019) and in this study.
